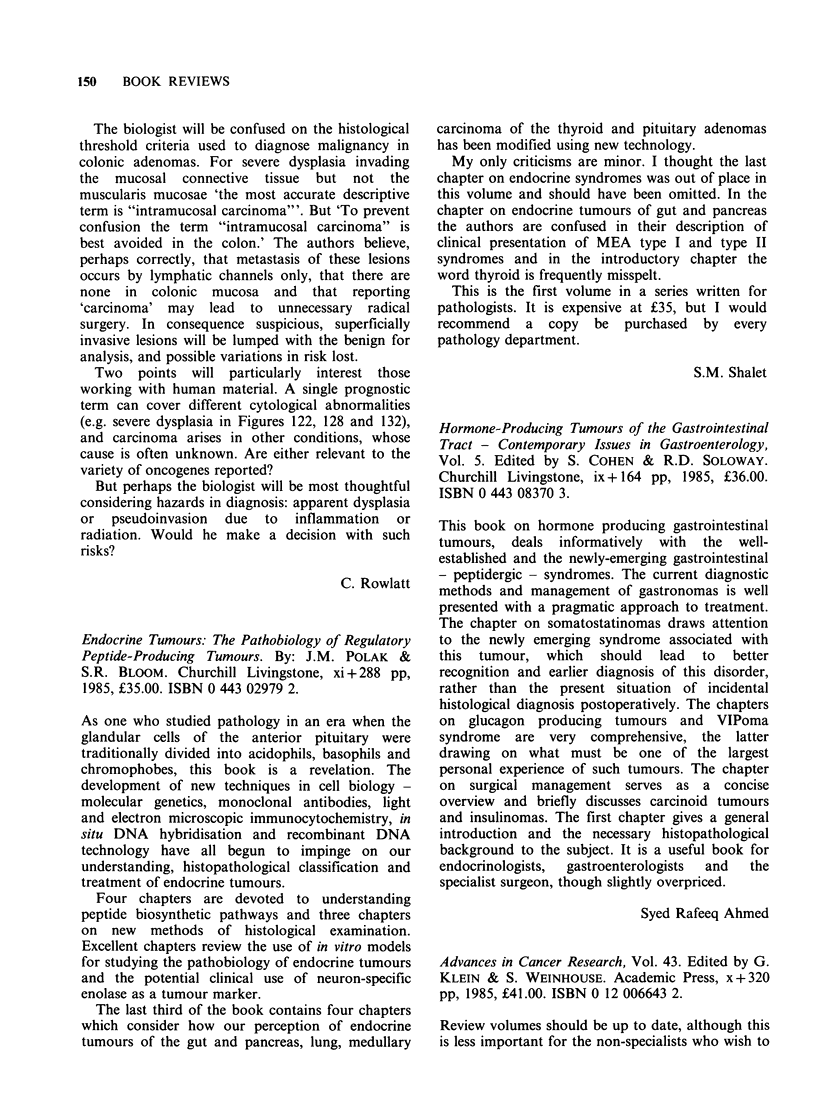# Endocrine Tumours: The Pathobiology of Regulatory Peptide-Producing Tumours

**Published:** 1986-01

**Authors:** S.M. Shalet


*Endocrine Tumours: The Pathobiology of Regulatory Peptide-Producing Tumours.* By: J.M. POLAK & S.R. BLOOM. Churchill Livingstone, xi+288 pp, 1985, £35.00. ISBN 0 443 02979 2.

As one who studied pathology in an era when the glandular cells of the anterior pituitary were traditionally divided into acidophils, basophils and chromophobes, this book is a revelation. The development of new techniques in cell biology – molecular genetics, monoclonal antibodies, light and electron microscopic immunocytochemistry, *in situ* DNA hybridisation and recombinant DNA technology have all begun to impinge on our understanding, histopathological classification and treatment of endocrine tumours.

Four chapters are devoted to understanding peptide biosynthetic pathways and three chapters on new methods of histological examination. Excellent chapters review the use of *in vitro* models for studying the pathobiology of endocrine tumours and the potential clinical use of neuron-specific enolase as a tumour marker.

The last third of the book contains four chapters which consider how our perception of endocrine tumours of the gut and pancreas, lung, medullary carcinoma of the thyroid and pituitary adenomas has been modified using new technology.

My only criticisms are minor. I thought the last chapter on endocrine syndromes was out of place in this volume and should have been omitted. In the chapter on endocrine tumours of gut and pancreas the authors are confused in their description of clinical presentation of MEA type I and type II syndromes and in the introductory chapter the word thyroid is frequently misspelt.

This is the first volume in a series written for pathologists. It is expensive at £35, but I would recommend a copy be purchased by every pathology department.

S.M. Shalet